# Supplementary material for: Priming mobilization of hair follicle stem cells triggers permanent loss of regeneration after alkylating chemotherapy
Source: Nat Commun. 2019 Aug 27;10:3694. doi: 10.1038/s41467-019-11665-0 (PMC6711970; doi:10.1038/s41467-019-11665-0)
Supplement: Supplementary file 1 — Supporting Information [file 41467_2019_11665_MOESM1_ESM.pdf]

Priming mobilization of hair follicle stem cells triggers permanent loss of regeneration after alkylating chemotherapy

Kim et al.

Supplementary Information

Supplementary Figures 1-12

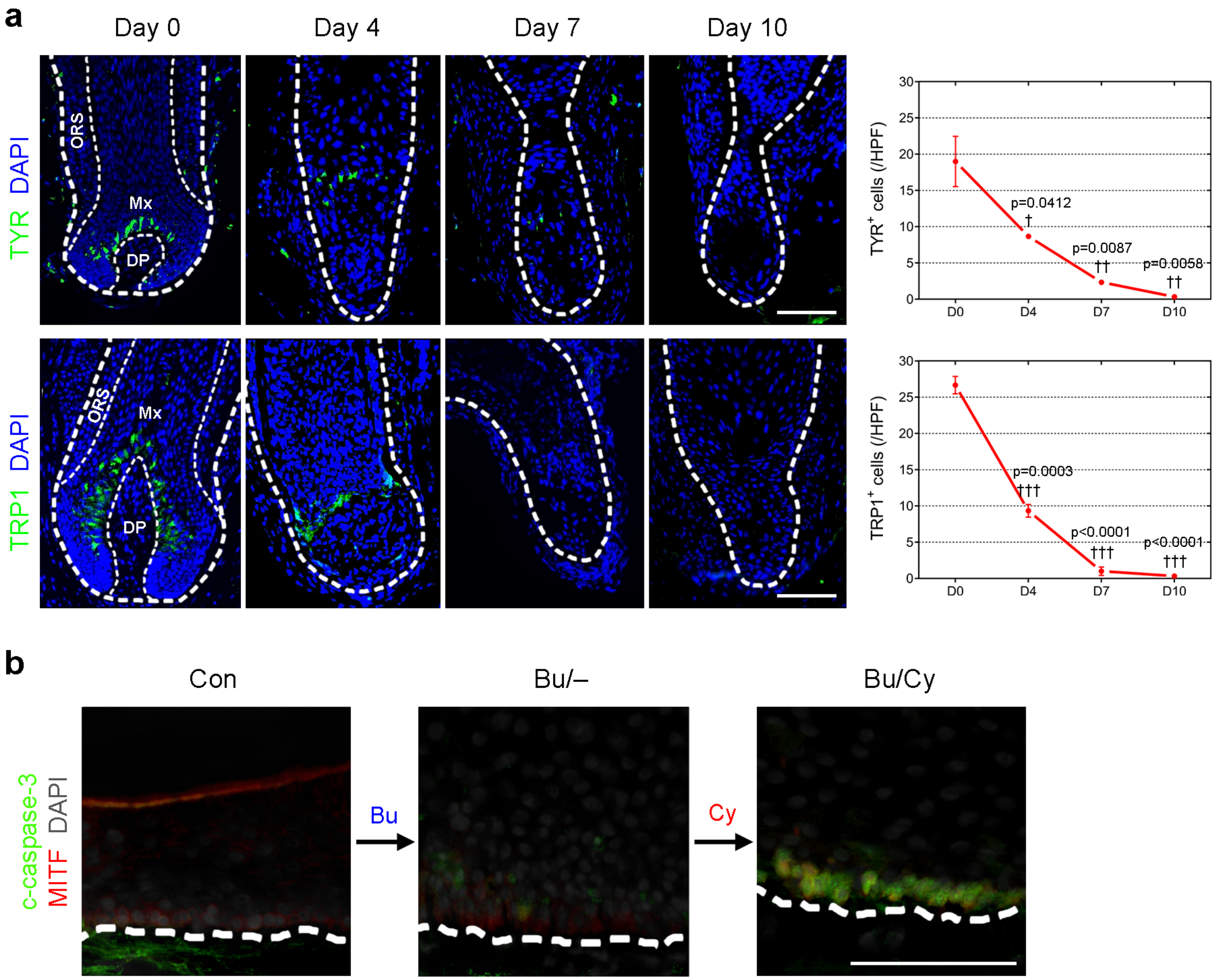

Supplementary Fig. 1. Loss of follicular melanocytes after Bu/Cy treatment

a, Representative images and quantification of TYR and TRP1 staining to detect follicular melanocytes in the bulb after Bu/Cy treatment (*n* = 3 biological replicates/timepoint). The numbers of TYR^+ and $TRP1^+$ cells started to decrease after Bu treatment (day 0 to 4) and disappeared after Bu/Cy treatment (day 4 to 7) in the bulb. **b,** Representative images of MITF and cleaved caspase-3 costaining to detect apoptosis of undifferentiated melanocytes in the bulge (immunofluorescence; scale bar = 100 μm).

Bu, busulfan; Cy, cyclophosphamide; Bu/Cy, busulfan followed by cyclophosphamide; HF, hair follicle; HPF, high-power field; Mx, hair matrix; ORS, outer root sheath; DP, dermal papilla; TYR, tyrosinase; TRP1, tyrosinase-related protein 1; MITF, melanocyte-inducing transcription factor; c-caspase-3, cleaved caspase-3. Data are mean ± SEM. Source data are provided as a Source Data file. $^{\dagger}p < 0.05$ (vs D0); $^{\dagger\dagger}p < 0.01$ (vs D0); $^{\dagger\dagger\dagger}p < 0.001$ (vs D0, unpaired *t* test).

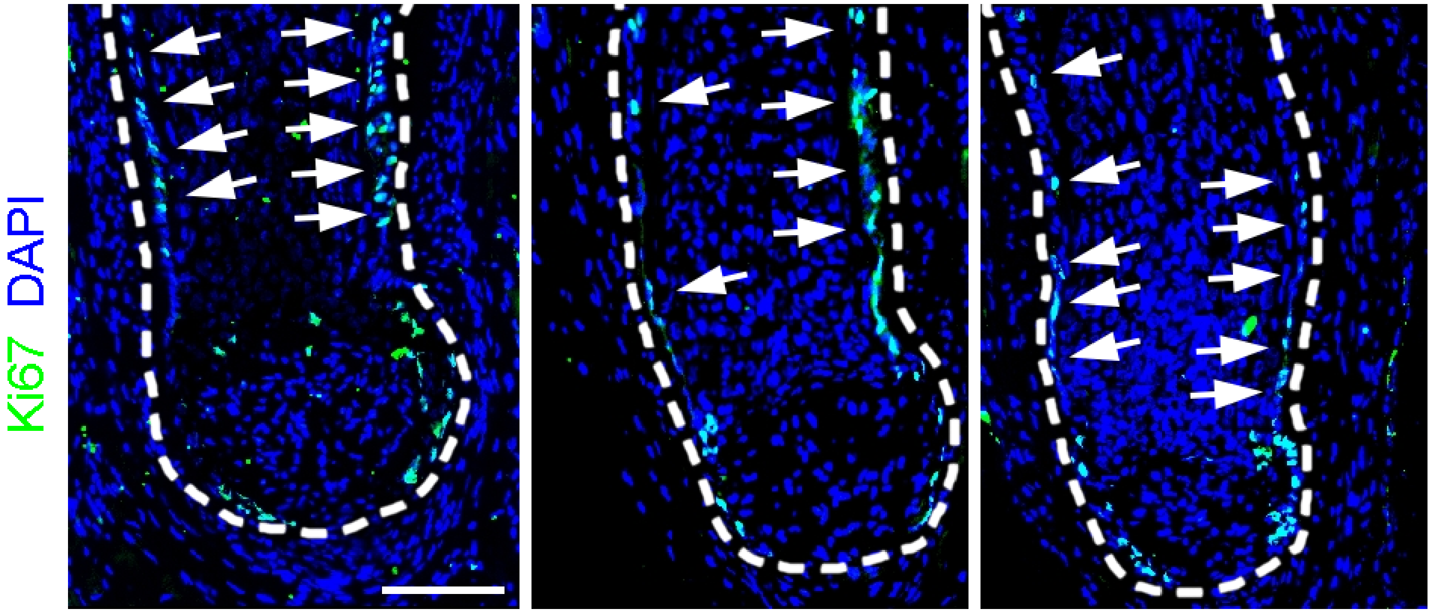

Supplementary Fig. 2. Bu-induced proliferation of basal ORS cells

Immunofluorescence images of Ki67 staining to detect cellular proliferation after Bu treatment. Basal ORS cells showed remarkable proliferation after Bu treatment (white arrow; on day 4). (immunofluorescence; scale bar = 100 μm).

Bu, busulfan; ORS, outer root sheath.

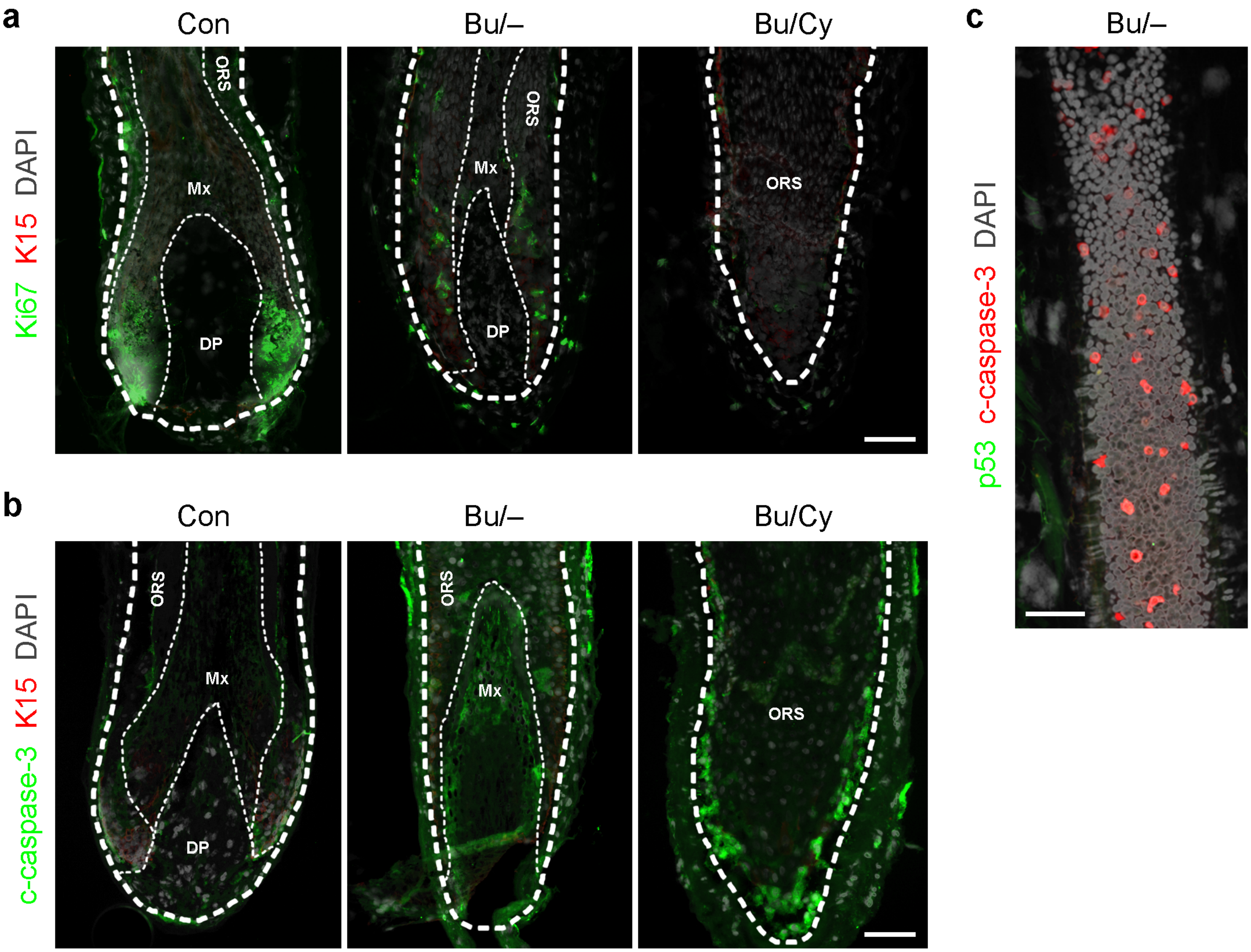

Supplementary Fig. 3. Spatiotemporal response in the bulb of organ-cultured HFs

Representative image of **a,** Ki67$^+$K15$^+$ cells and **b,** c-caspase-3$^+$K15$^+$ cells in the bulb of organ-cultured HFs after Bu/Cy treatment and **c,** p53$^+$c-caspase-3$^+$ cells in the epithelial strands of organ-cultured HFs after Bu treatment. Bu-treated HFs prematurely entered catagen stage without p53-dependent apoptosis in their epithelial strands (immunofluorescence; scale bar = 100 μm).

Bu, busulfan; Bu/Cy, busulfan followed by cyclophosphamide; HF, hair follicle; Mx, hair matrix; ORS, outer root sheath; DP, dermal papilla; c-caspase-3, cleaved caspase-3.

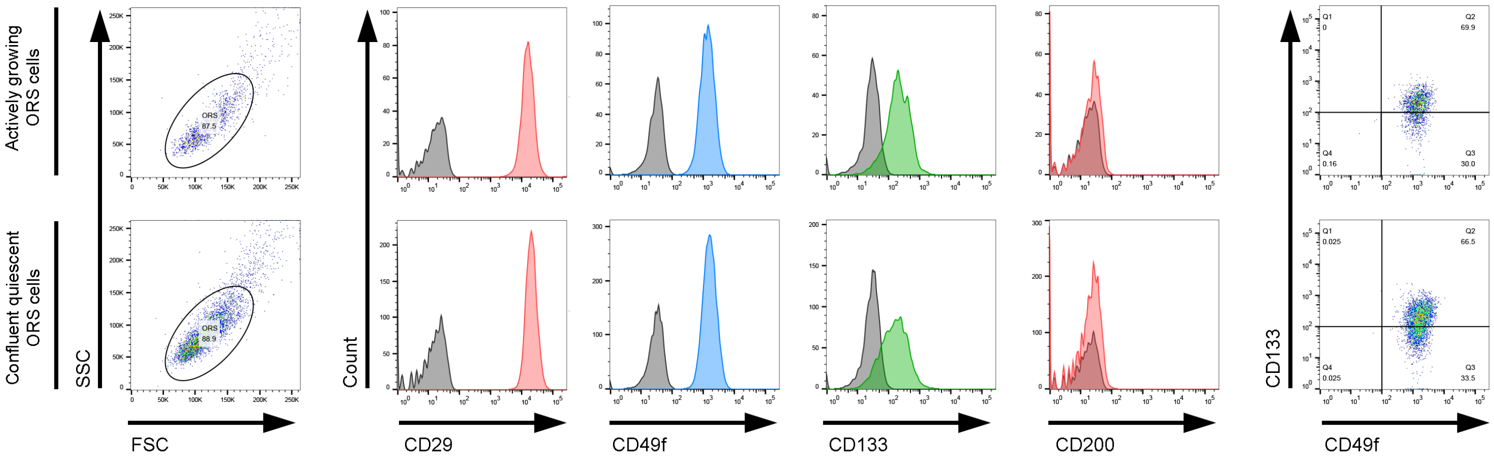

Supplementary Fig. 4. Comparison of actively growing and confluent quiescent ORS cells

Representative flow cytometric analysis to compare surface markers in the actively growing and confluent quiescent ORS cell populations. Both populations showed homogeneous characteristics except for the proportion of cells in S phase.

ORS, outer root sheath.

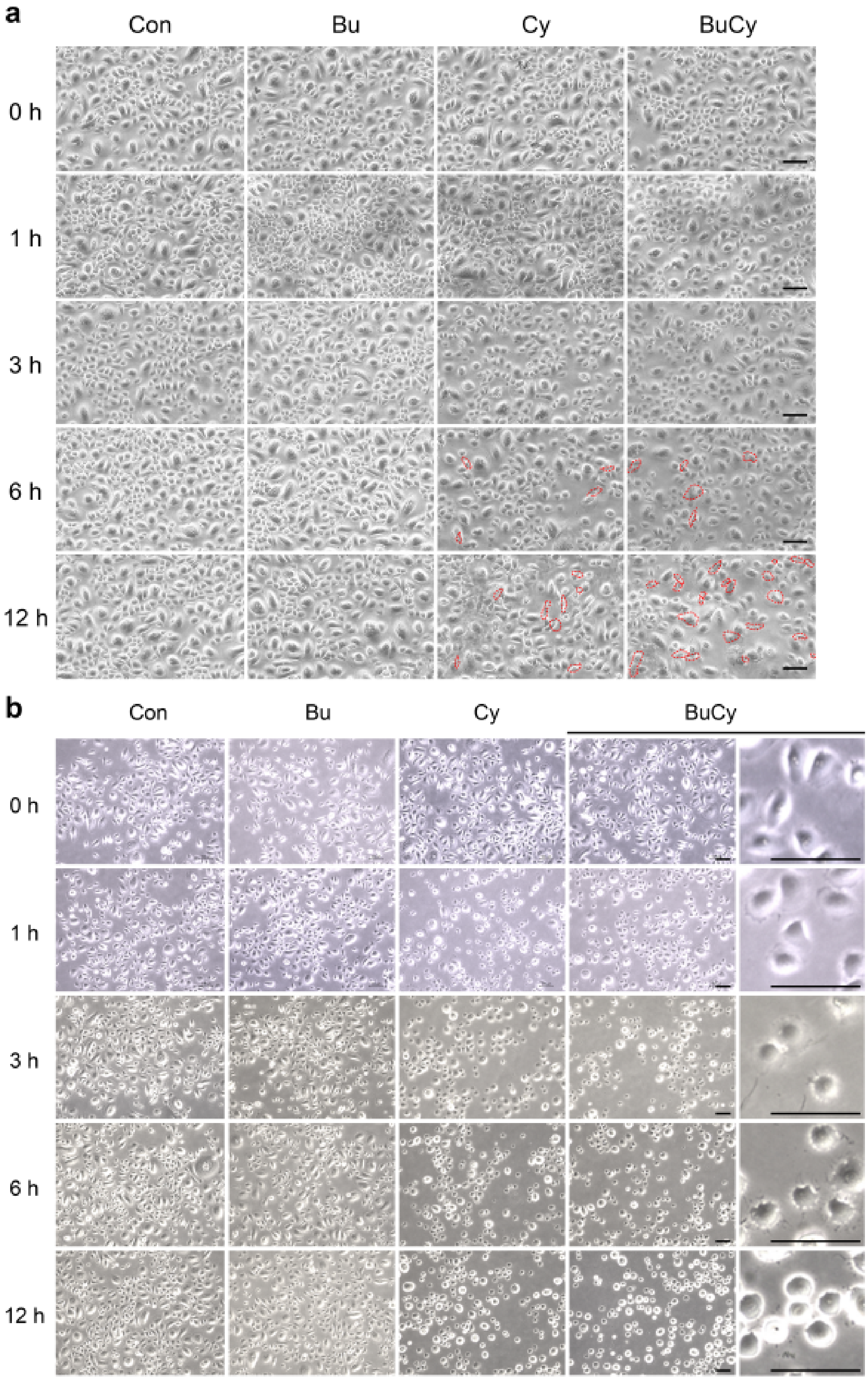

Supplementary Fig. 5. Morphological changes in human ORS cells after Bu and/or Cy treatment Representative microscopic images showing the morphological changes in holoclone-rich ORS cells after 1, 3, 6, and 12 h of Bu and/or Cy treatment, **a,** 100% confluency vs **b,** 70-80% confluence. The floating dead cells were distinguished by their free movement in medium (red-dotted line). The Cy-treated cells showed gradual changes, including membrane undulation, cellular shrinkage, and ultimately, detachment from and floating in the dish (scale bar = 50 μm). Bu, busulfan; Cy, cyclophosphamide; ORS, outer root sheath.

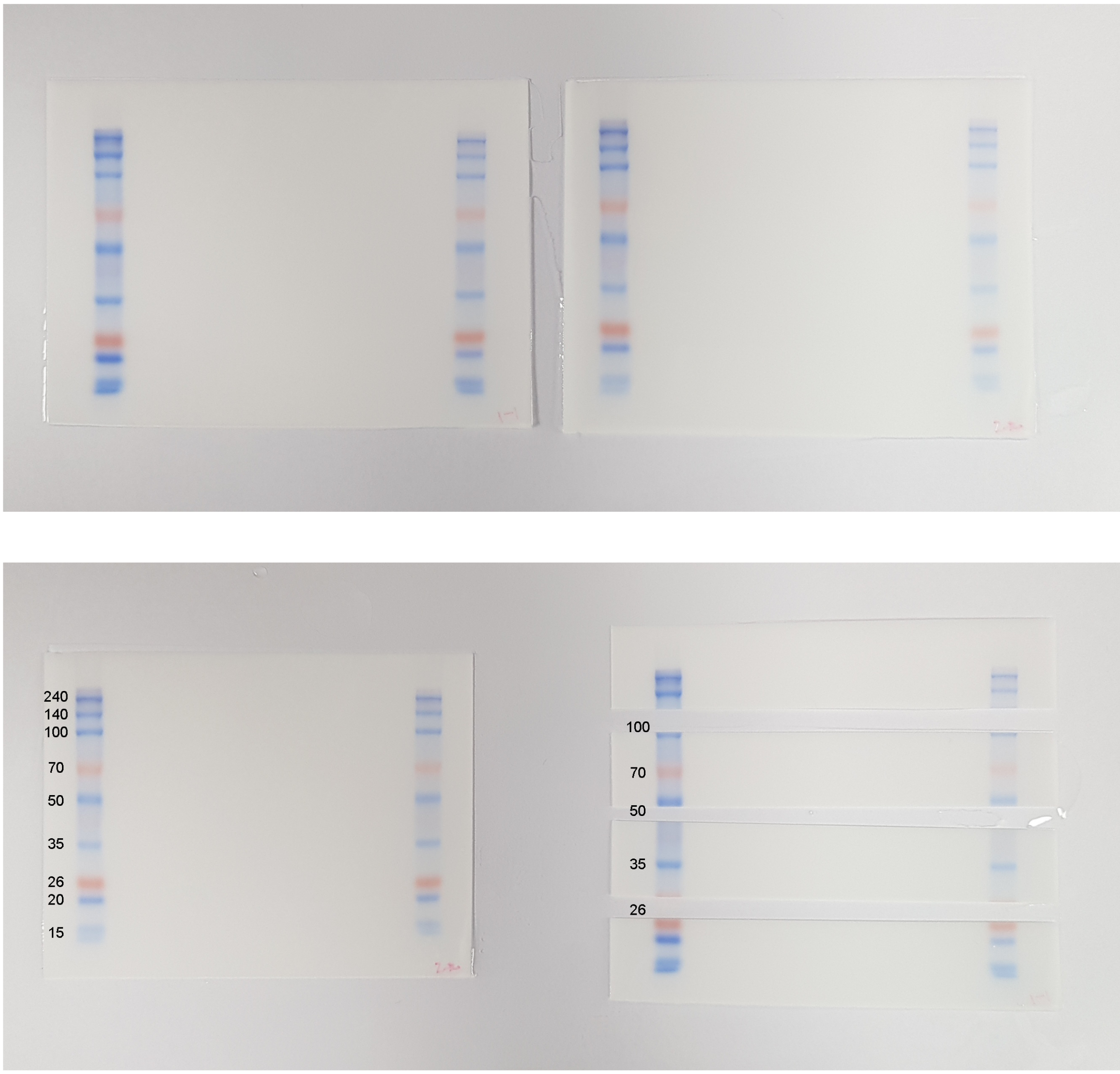

Supplementary Fig. 6. Membrane cutting for western blot analysis

The blotted membranes were cut horizontally according to the size marker and were incubated with the primary antibodies corresponding to the target protein size.

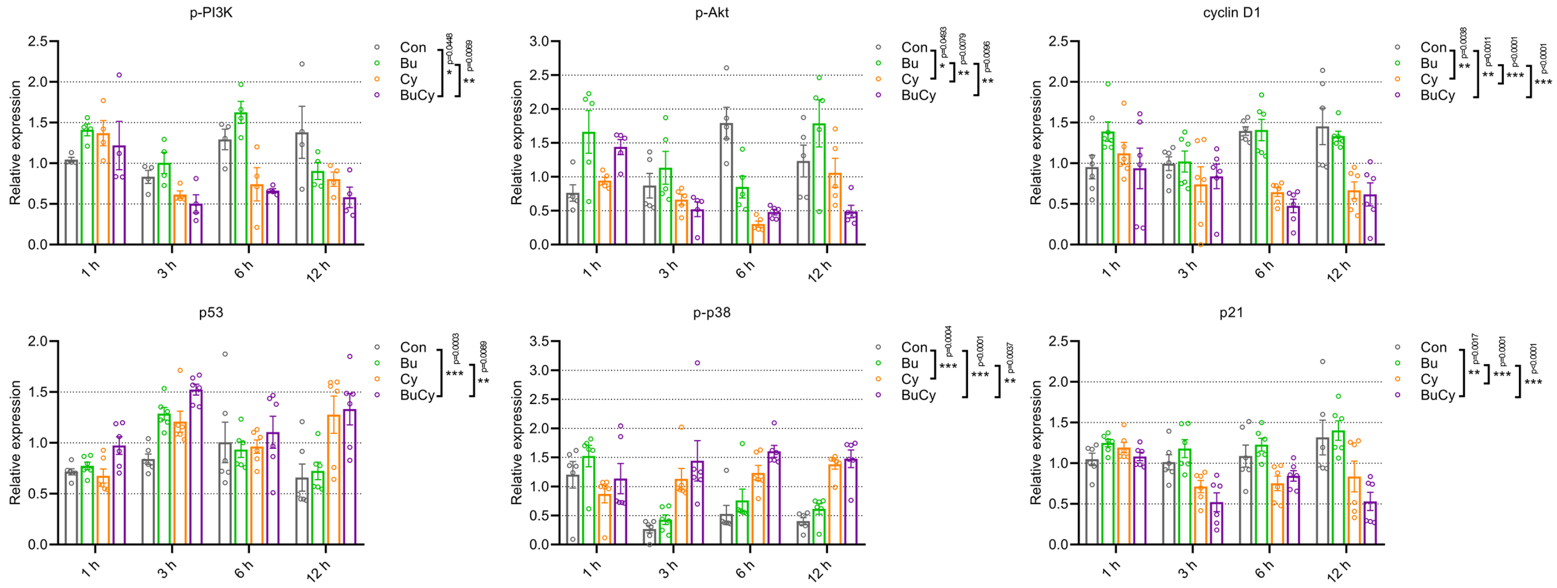

Supplementary Fig. 7. Bu and Cy trigger key signaling pathways for proliferation and cell death.

Quantification plots of protein analysis after Bu and/or Cy treatment comparing the concentrations of phosphorylated PI3K (*n* = 4), phosphorylated Akt (*n* = 5), cyclin D1 (*n* = 6), p53 (*n* = 6), phosphorylated p38 (*n* = 6), and p21 (*n* = 6), with β-actin as a loading control (6 biological samples).

Bu, busulfan; Cy, cyclophosphamide.

Data are mean ± SEM. Source data are provided as a Source Data file. *$p < 0.05$; **$p < 0.01$; ***$p < 0.001$ (two-way analysis of variance with Tukey's multiple comparisons test).

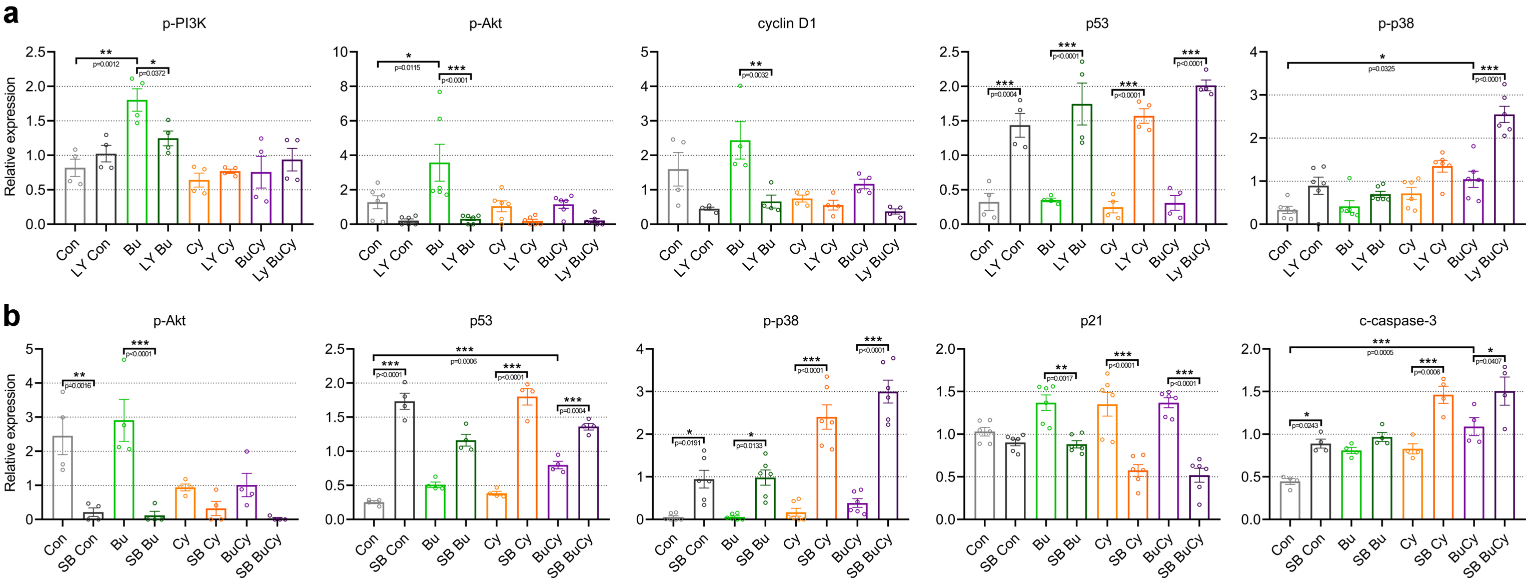

Supplementary Fig. 8. Roles of PI3K/Akt and p53/p38 in phase conversion after sequential Bu/Cy treatment

a, Quantification plots of protein analysis after Bu and/or Cy treatment with PI3K inhibitor (20 μM; LY294002) comparing the concentrations of phosphorylated PI3K (*n* = 4), phosphorylated Akt (*n* = 6), cyclin D1 (*n* = 4), p53 (*n* = 4), and phosphorylated p38 (*n* = 6), with β-actin as a loading control (4 biological samples). **b,** Quantification plots of protein analysis after Bu and/or Cy treatment with p38 inhibitor (10-20 μM; SB202190 or SB203580) comparing the concentrations of phosphorylated Akt (*n* = 4), p53 (*n* = 4), phosphorylated p38 (*n* = 6), p21 (*n* = 6), and cleaved caspase-3 (*n* = 4), with β-actin as a loading control (4 biological samples).

Bu, busulfan; Cy, cyclophosphamide; LY, LY294002; SB, SB202190 or SB203580; c-caspase-3, cleaved caspase-3.

Data are mean ± SEM. Source data are provided as a Source Data file. *$p < 0.05$; **$p < 0.01$; ***$p < 0.001$ (one-way analysis of variance with Tukey's multiple comparisons test).

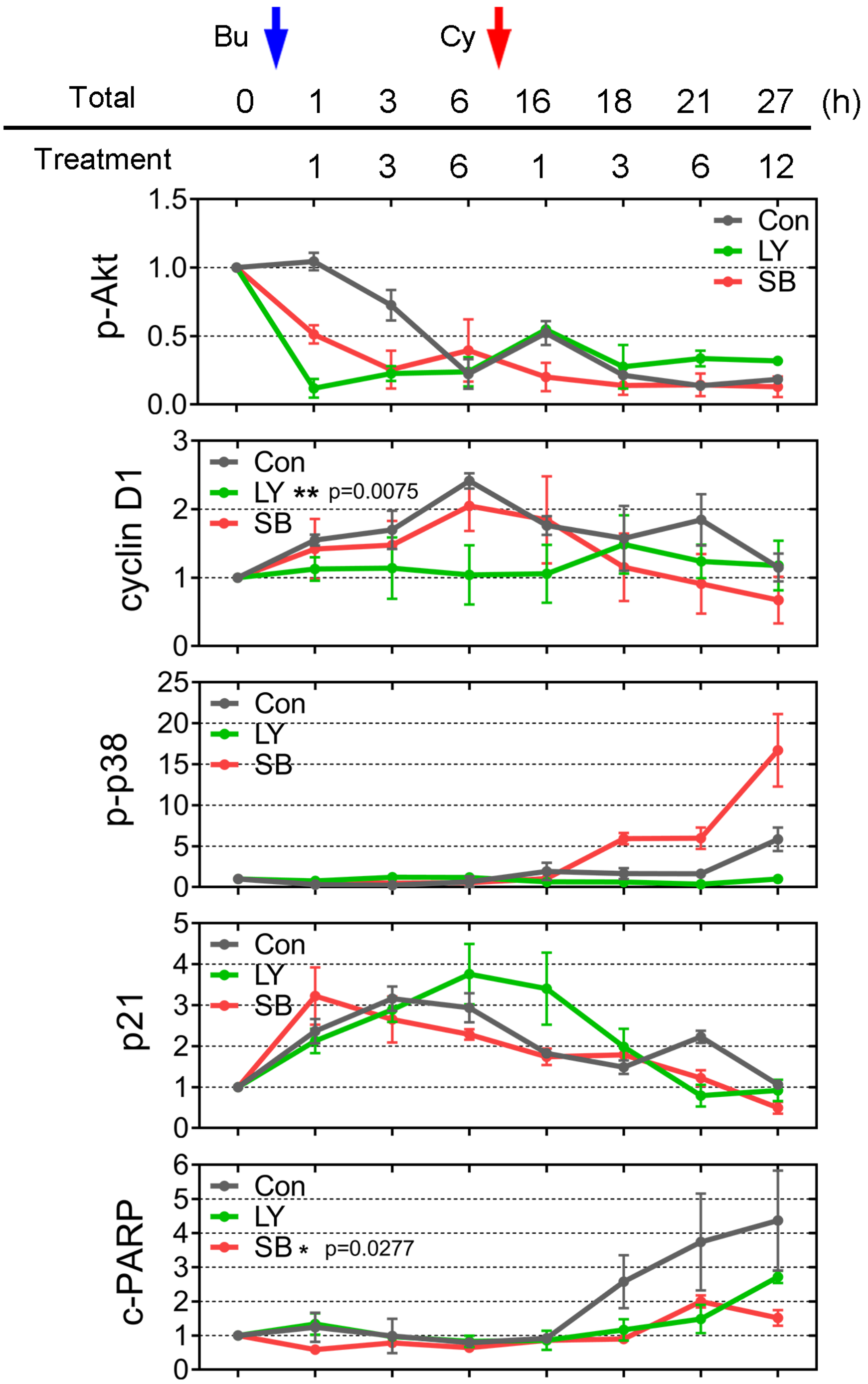

Supplementary Fig. 9. Roles of Akt and p38 in phase conversion after sequential Bu/Cy treatment

Temporal quantification plots of protein analysis after sequential Bu/Cy treatment with PI3K or p38 inhibitor of the concentrations of phosphorylated Akt, cyclin D1, phosphorylated p38, p21, and cleaved PARP, with β-actin as a loading control (n = 4 biological replicates).

Bu, busulfan; Cy, cyclophosphamide; LY, LY294002; SB, SB202190 or SB203580; c-PARP, cleaved PARP.

Data are mean ± SEM. Source data are provided as a Source Data file. $*p < 0.05$ (vs Con); $**p < 0.01$ (vs Con, two-way analysis of variance with Dunnett's multiple comparisons test).

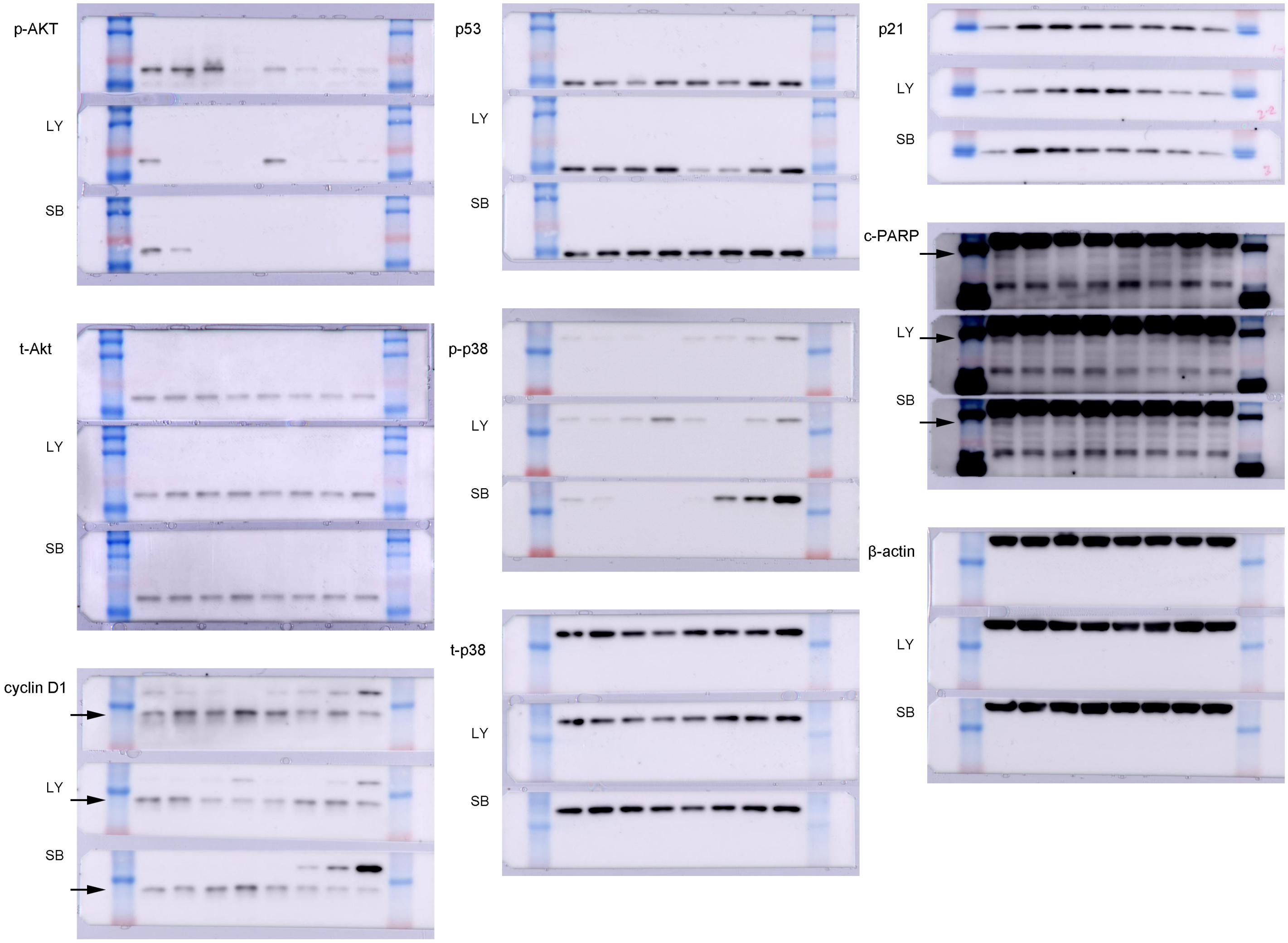

Supplementary Fig. 10. Roles of PI3K/Akt and p53/p38 in phase conversion after Bu/Cy treatment

Representative blots for protein analysis after sequential Bu/Cy treatment with PI3K or p38 inhibitor showing the temporal changes of phosphorylated and total Akt, cyclin D1, p53, phosphorylated and total p38, p21, and cleaved PARP, with β-actin as a loading control.

Bu, busulfan; Cy, cyclophosphamide; Bu/Cy, busulfan followed by cyclophosphamide; LY, LY294002; SB, SB202190 or SB203580; c-PARP, cleaved PARP.

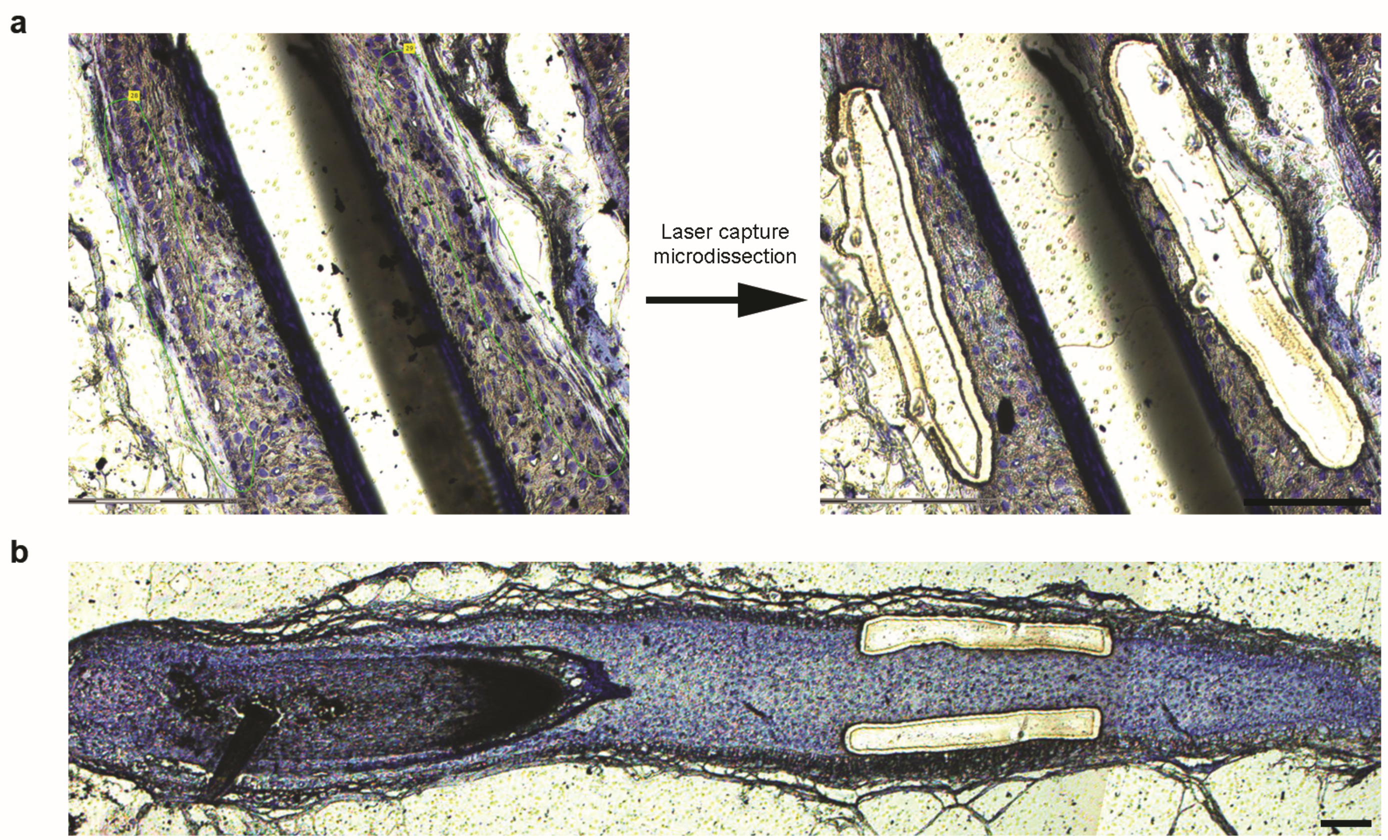

Supplementary Fig. 11. Laser capture microdissection of longitudinal sections of the *in vivo* HF xenografts Representative microscopic images of the basal layer in the bulge of human HFs. **a,** The basal bulge cells were catapulted into an AdhesiveCap (Carl Zeiss) positioned above the section. **b,** The bulge area was identified in longitudinal cryosections of the *in vivo* HF xenografts (cresyl violet stain, scale bar = 100 µm).

HF, hair follicle; ORS, outer root sheath.

a Pathway networks upregulated after Bu/— treatment

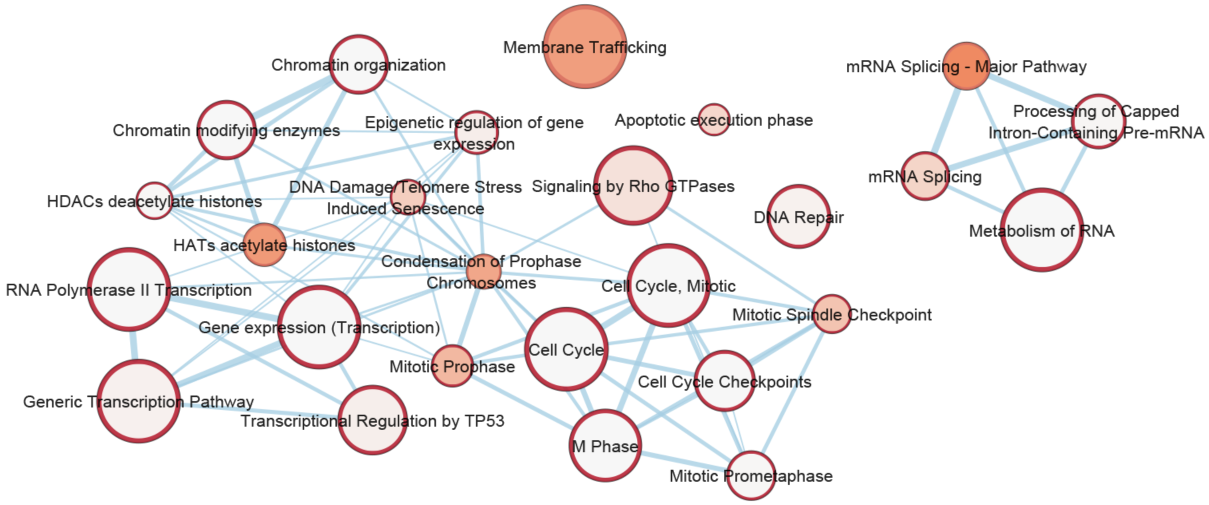

b Pathway networks upregulated after Bu/Cy treatment

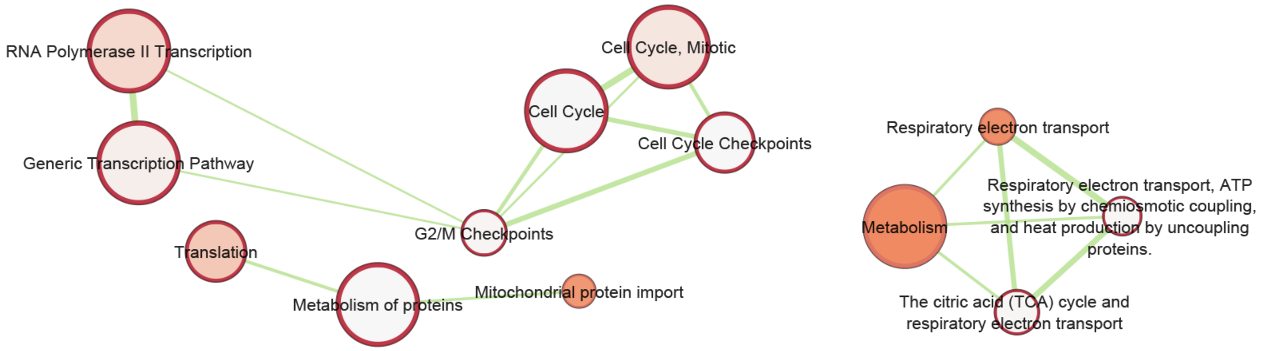

Supplementary Fig. 12. Enrichment pathway network from the Reactome database

a, Mitotic cell cycle pathways were substantially upregulated after Bu treatment, accompanied by the DNA repair pathway and p53 transcriptional regulation. **b,** Cell cycle checkpoint pathways, especially the G2/M checkpoint, were upregulated after Bu/Cy treatment, along with mitochondrial membrane-associated pathways (adjusted p value < 0.01).

Bu, busulfan; Cy, cyclophosphamide; Bu/Cy, busulfan followed by cyclophosphamide.
