# Supplementary material for: Priming mobilization of hair follicle stem cells triggers permanent loss of regeneration after alkylating chemotherapy
Source: Nat Commun. 2019 Aug 27;10:3694. doi: 10.1038/s41467-019-11665-0 (PMC6711970; doi:10.1038/s41467-019-11665-0)
Supplement: Supplementary file 3 — Reporting Summary [file 41467_2019_11665_MOESM3_ESM.pdf]

nature research

Corresponding author(s): Ohsang Kwon

Last updated by author(s): Jul 14, 2019

Reporting Summary

Nature Research wishes to improve the reproducibility of the work that we publish. This form provides structure for consistency and transparency in reporting. For further information on Nature Research policies, see Authors & Referees and the Editorial Policy Checklist.

Statistics

For all statistical analyses, confirm that the following items are present in the figure legend, table legend, main text, or Methods section.

n/a	Confirmed	
☐	☒	The exact sample size (*n*) for each experimental group/condition, given as a discrete number and unit of measurement
☐	☒	A statement on whether measurements were taken from distinct samples or whether the same sample was measured repeatedly
☐	☒	The statistical test(s) used AND whether they are one- or two-sided<br>*Only common tests should be described solely by name; describe more complex techniques in the Methods section.*
☐	☒	A description of all covariates tested
☐	☒	A description of any assumptions or corrections, such as tests of normality and adjustment for multiple comparisons
☐	☐	A full description of the statistical parameters including central tendency (e.g. means) or other basic estimates (e.g. regression coefficient) AND variation (e.g. standard deviation) or associated estimates of uncertainty (e.g. confidence intervals)
☐	☒	For null hypothesis testing, the test statistic (e.g. *F*, *t*, *r*) with confidence intervals, effect sizes, degrees of freedom and *P* value noted<br>*Give P values as exact values whenever suitable.*
☒	☐	For Bayesian analysis, information on the choice of priors and Markov chain Monte Carlo settings
☐	☒	For hierarchical and complex designs, identification of the appropriate level for tests and full reporting of outcomes
☒	☐	Estimates of effect sizes (e.g. Cohen's *d*, Pearson's *r*), indicating how they were calculated

Our web collection on statistics for biologists contains articles on many of the points above.

Software and code

Policy information about availability of computer code

Data collection	FACSDiva software (BD Biosciences)<br>Amersham Imager 680 (GeHealthcare, Chicago, IL, USA)<br>Illumina HiSeq 2500 sequencer (Illumina)
Data analysis	GraphPad Prism v8.0 (GraphPad Software)<br>ModFit LT software (BD Biosciences)<br>FlowJo v10 software (FlowJo, LLC)<br>ImageJ software (National Institutes of Health)<br>RSEM-1.3.0<br>the R package edgeR<br>the R package pheatmap<br>g:Profiler online tool<br>Cytoscape software (National Resource for Network Biology)<br>BioRender online flatform

For manuscripts utilizing custom algorithms or software that are central to the research but not yet described in published literature, software must be made available to editors/reviewers. We strongly encourage code deposition in a community repository (e.g. GitHub). See the Nature Research guidelines for submitting code & software for further information.

October 2018

Data

Policy information about availability of data

All manuscripts must include a data availability statement. This statement should provide the following information, where applicable:

- Accession codes, unique identifiers, or web links for publicly available datasets
- A list of figures that have associated raw data
- A description of any restrictions on data availability

Authors can confirm that all relevant data are included as "Source Data" in the main manuscript, supplementary information, NCBI GEO website (accession GEO GSE130054).

Field-specific reporting

Please select the one below that is the best fit for your research. If you are not sure, read the appropriate sections before making your selection.

☒ Life sciences ☐ Behavioural & social sciences ☐ Ecological, evolutionary & environmental sciences

For a reference copy of the document with all sections, see nature.com/documents/nr-reporting-summary-flat.pdf

Life sciences study design

All studies must disclose on these points even when the disclosure is negative.

Sample size	The sample size for experiment is indicated in the figure legends as the number of biological replicates and independent experiment. Sample sizes were chosen based the previous experimental data of the chemotherapy-induced responses and the previous experience for each experiment to yield high power to detect specific effects.
Data exclusions	No data was excluded from the analysis.<br>In the cell cycle analysis, some data point was unable to obtain due to excessive cell death.
Replication	Experiments were repeated so that our data are based on at least three or more biological replicates with independent experiments. The precise number of replication are given in the figure legend and the Method section.
Randomization	Human hair follicles from a healthy volunteer were randomly distributed into the experimental groups.
Blinding	Number of positive cells was counted with blinded image information regarding the experimental group.

Reporting for specific materials, systems and methods

We require information from authors about some types of materials, experimental systems and methods used in many studies. Here, indicate whether each material, system or method listed is relevant to your study. If you are not sure if a list item applies to your research, read the appropriate section before selecting a response.

Materials & experimental systems

n/a	Involved in the study	
☐	☒	Antibodies
☒	☐	Eukaryotic cell lines
☒	☐	Palaeontology
☐	☒	Animals and other organisms
☐	☒	Human research participants
☒	☐	Clinical data

Methods

n/a	Involved in the study	
☒	☐	ChIP-seq
☐	☒	Flow cytometry
☒	☐	MRI-based neuroimaging

Antibodies

Antibodies used	For immunohistochemistry, following antibody was used after dilution in antibody diluent reagent: p53 (SC126, 1:50; Santa Cruz Biotechnology).<br>For immunofluorescence, following antibodies were used after dilution in antibody diluent reagent: Fas (ab82419, 1:100; Abcam, MA, USA), Ki67 (SP6, 1:200; Spring Bioscience, Pleasanton, CA, USA or MIB-1, 1:200; Dako), K15 (LHK15, 1:200; Thermo Fisher), p53 (DO-7, 1:800; Novocastra, Newcastle, England or 1C12, 1:1000; Cell Signaling, Danvers, MA, USA), K14 (GTX104124, 1:1000; GeneTex, Irvine, CA, USA), cleaved caspase-3 (5A1E, 1:400; Cell Signaling), γ-H2AX (#2577, 1:800; Cell Signaling), Shh (EP1190Y, 1/500; Abcam), Lhx2 (C-20, 1:200; Santa Cruz Biotechnology), integrin β4 (H-101, 1:200; Santa Cruz Biotechnology), anti-phospho Akt (#9275, 1:800; Cell Signaling), anti-phospho p38 (#9211, 1:800; Cell Signaling), TYR (T311, 1:100; Thermo Fisher, Waltham, MA, USA), TRP1 (SC25543, 1:100; Santa Cruz Biotechnology), MITF (C5, 1:100; Thermo Fisher), and Alexa Fluor 488/594-labeled goat IgG antibody (1:200, Thermo Fisher).

October 2018

	For western blot, following primary antibodies were used: anti-phospho PI3K (#4228, 1:1000; Cell Signaling), anti-phospho Akt (#9275, 1:1000; Cell Signaling), anti-total Akt (#9272, 1:1000; Cell Signaling), anti-cyclin D1 (#2926, 1:2000; Cell Signaling), anti-p53 (#2524, 1:1000; Cell Signaling), anti-phospho p38 (#9211, 1:1000; Cell Signaling), anti-total p38 (#9212, 1:1000; Cell Signaling), anti-p21 (#2947, 1:1000; Cell Signaling), anti-cleaved caspase-3 (#9664, 1:1000; Cell Signaling), anti-PARP (#9542, 1:1000; Cell Signaling) and anti-β-actin (SC-1616, 1:1000; Santa Cruz Biotechnology or BA3R, 1:5000; Thermo Fisher).
Validation	Validation is available on the manufacturer's website.

Animals and other organisms

Policy information about studies involving animals; ARRIVE guidelines recommended for reporting animal research

Laboratory animals	Microdissected human hair follicles were transplanted into 7-week-old, female, severe combined immunodeficiency hairless outbred mice (SHO mice; 18–20 g; Jackson Laboratory, ME, USA).
Wild animals	No wild animals were used.
Field-collected samples	No field-collected samples were used.
Ethics oversight	All animal procedures were performed according to the American Association for the Accreditation of Laboratory Animal Care guidelines and approved by the Institutional Animal Care and Use Committee of the Seoul National University Hospital (approval number 12-0066).

Note that full information on the approval of the study protocol must also be provided in the manuscript.

Human research participants

Policy information about studies involving human research participants

Population characteristics	Human scalp samples (2 × 1.5 cm) were obtained from the occipital scalp of 14 healthy male volunteers (37–55 years old) who did not have any current or prior scalp disease.
Recruitment	Healthy male volunteers were randomly recruited based on the public advertisement.
Ethics oversight	This study was approved by the Institutional Review Board of the Seoul National University Hospital (approval number 1310-075-527). All human protocols conformed to the ethical principles of the Declaration of Helsinki, and written informed consent was obtained from all human subjects.

Note that full information on the approval of the study protocol must also be provided in the manuscript.

Flow Cytometry

Plots

Confirm that:

☒ The axis labels state the marker and fluorochrome used (e.g. CD4-FITC).

☒ The axis scales are clearly visible. Include numbers along axes only for bottom left plot of group (a 'group' is an analysis of identical markers).

☒ All plots are contour plots with outliers or pseudocolor plots.

☒ A numerical value for number of cells or percentage (with statistics) is provided.

Methodology

Sample preparation	Sample preparation is described in Methods
Instrument	FACSCalibur™ instrument
Software	FACSDiva software for collection and ModFit LT and FlowJo v10 softwares for analysis
Cell population abundance	Purity was determined by relevant staining using flow cytometry. 10,000 cellular events were originally collected. In case of the BuCy or Bu/Cy-treated cells, it is impossible to obtain 10,000 cellular events due to excessive cell death.
Gating strategy	Control stains (unstained cells) were used to set gates. FSC-A/SSC-A gates of the starting cell population were used to discriminate between viable cells and cell debris, followed by FSC-A/FSC-H gating to select singlet cells.

☒ Tick this box to confirm that a figure exemplifying the gating strategy is provided in the Supplementary Information.
